# Malignant Diffuse-Type Tenosynovial Giant Cell Tumor in the Subcutaneous Tissue of the Midthigh: A Report of a Rare Tumor in an Unusual Location

**DOI:** 10.1155/2022/6986741

**Published:** 2022-09-19

**Authors:** Omar Salem, Khalid Kurdi, Amani Joudeh, Ahmad Al-Dhafiri, Zahra Alkhunaizi, Emad Al Absi

**Affiliations:** ^1^Department of Orthopedics, King Fahad Specialist Hospital, Saudi Arabia; ^2^Department of Pathology and Laboratory Medicine, King Fahad Specialist Hospital, Saudi Arabia; ^3^Department of Medical Imaging, King Fahad Specialist Hospital, Saudi Arabia

## Abstract

Malignant TS-GCT is an extremely rare and aggressive tumor with only few cases published in the literature, due to the small number of cases is not completely understood and is diagnostically challenging. Although surgical treatment is the primary treatment modality, there is no consensus regarding adjuvant treatment. Regardless of mode of treatment, the tumor still caries unfavorable prognosis. In this paper, we reviewed the literature for cases of malignant TS-GCT. We also would like to present an additional case of malignant TS-GCT that was found in an unusual location in subcutaneous tissue of the midthigh.

## 1. Introduction

Tenosynovial giant cell tumors (TS-GCT) are common soft tissue tumors that originates from synovium of joints, tendons, and bursae [1]. On extreme rare occasions, malignant transformation has been found. Malignant TS-GCT are mostly located in the periarticular soft tissue of the lower extremity. Only few cases are found to be remote from the articular area. In this article, we would like to share our experience with a 34-year-old female with subcutaneous malignant TS-GCT of the right midthigh. Presence of such lesions in such unusual location could be misleading and diagnostically challenging. We also would like to point common clinical, radiological, and histological features that was observed while reviewing the literature.

## 2. Case Presentation

**Figure 1:**
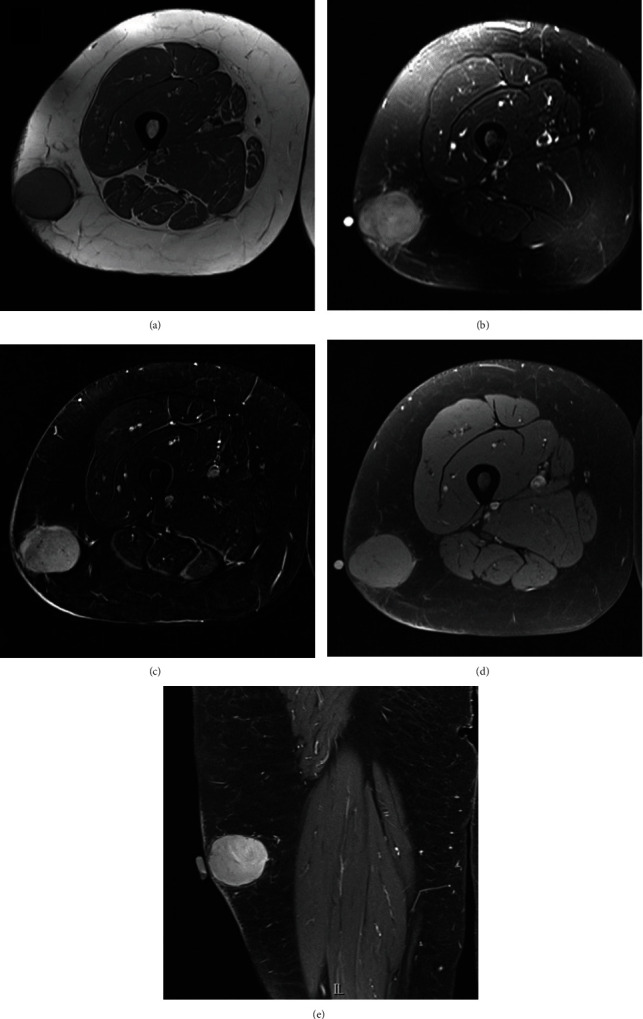
MRI (a) axial T1 WI nonfat saturated demonstrates mild hyperintensity compared to skeletal muscles. (b) Axial T2 WI with fat saturation demonstrates marked homogenous hyperintensity. (c) Axial subtraction image (pre- and post-IV gadolinium contrast) T1 WI with fat saturation confirms the homogeneous avid enhancement. (d) Axial T1 WI with fat saturation, precontrast demonstrates mild hyperintensity compared to skeletal muscles. No fat signal suppression. (e) Coronal T1 WI with fat saturation, post-IV gadolinium contrast injection demonstrates homogeneous avid enhancement.

**Figure 2:**
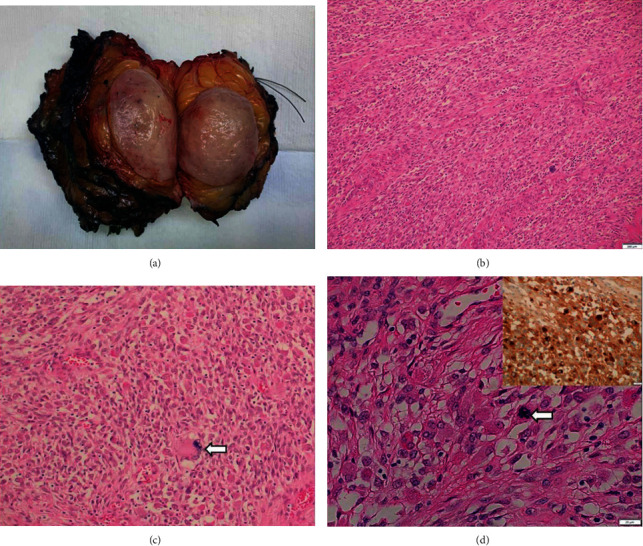
The tumor grossly shows fleshy gray cut surface with well-circumscribed round edges (a). Histological examination shows nested pseudoalveolar proliferation of histiocytes and synoviocytes showing abundant eosinophilic cytoplasm with some perinuclear hemosiderin (H&E, (b) -100x and (c) -200x). Scattered multinucleated osteoclast-like giant cells are seen (H&E (c) -200x, arrow). The tumor shows atypical cytological features in the form of atypical mitotic figures (H&E (d) -400x, arrow). The neoplastic cells are diffusely positive for immunohistochemical stain for clusterin antibody (clusterin IHC, (e) -upper right insert).

Our case is a 34-year-old female who presented with a slowly growing painless mass in the right midthigh over a year. It was not painful and was not interfering with her activity. Clinical examination showed 5 × 4 cm mass in the lateral aspect of the midthigh. The mass was superficial to the underlying muscles and fascia. MRI showed a well-defined oval shaped soft tissue mass in the superficial subcutaneous tissue of the right proximal thigh (Figure 1). The lesion is measuring 3.5 × 4.4 × 3.8 cm. It was separated from the lateral and posterior compartment thigh muscles and fascia by a clear fat plane. The mass was homogenous T2 hyperintense as well as T1 signal intensity that is slightly higher than skeletal muscles. Postcontrast images show intense homogenous enhancement without necrosis. The mass was worrisome for soft tissue sarcoma so bedside tru-cut biopsy was done. Histological examination shows nested pseudoalveolar proliferation of histiocytes and synoviocytes showing abundant eosinophilic cytoplasm with some perinuclear hemosiderin. Scattered multinucleated osteoclast-like giant cells were seen. Our initial impression was benign diffused type tenosynovial giant cell tumor (Figure 2). Due to the aggressive behavior of such lesion, wide local excision was done with negative margins. Further histopathological analysis of the mass showed atypical cytological features, increasing cellularity and abundant eosinophilic cytoplasm in the histiocytoid cells which was consistent with a malignant process. The patient received postoperative adjuvant radiation therapy (PORTh 60Gy/30Fs) followed by adjuvant chemotherapy a total of 6 cycles of doxorubicin/dacarbazine. The patient is 14 months after the surgery, and she is disease free.

## 3. Discussion

TS-GCT are a group of benign soft tissue tumors that originates from the synovium of the joints, tendons, and bursae [1]. They were first described by Jaffe et al. in 1941. They are divided into two types localized and diffuse based on their growth pattern. Each can present either intra-articular or extra-articular. Due to its high proliferative index, diffuse-type TS-GCT has the propensity to be more aggressive and may therefore lead to joint impairment [1, 2]. However, both have a high tendency of local recurrence, with metastases being extremely rare.

Due to the polymorphic nature of TS-GCT, it was initially thought that it represents reactive inflammatory changes of the synovium. Since it malignantly transforms, they were then considered to be neoplastic in nature [3]. The identification of genetic abnormalities including rearrangement of 1p11–13 and the CSF1 locus in these tumors further supported the neoplastic hypothesis [1, 2].

Malignant TS-GCT tumor is extremely rare. They are believed to represent for less than 0.1% of benign TS-GCT [4]. It was first described by Castens and Howell in 1979. They described malignant recurrence of a previously benign TS-GCTof the foot [3]. According to Enzinger and Weiss, malignant TS-GCT is defined by the presence of sarcoma within or at a site of previously excised benign TS-GCT. They classified them into primary (De novo) and secondary (metachronous), primary when both benign TS-GCT occur with a coexisting sarcomatous component on initial presentation, while secondary presents as a sarcomatous recurrence of a previous beginning giant cell tumor of the tendon sheath [5]. The sarcomatous component of malignant TS-GCT was found to present in different forms including GCT-like, fibrosarcoma-like, MFH-like, myxofibrosarcoma-like, and osteosarcoma-like. GCT-like pattern was found to be the most common form. Several other lesions were found to share histological features with malignant TS-GCT including, primary carcinoma, metastatic melanoma, primary myxoid chondrosarcoma, osteosarcoma, and malignant fibrous histiocytoma. This makes histological identification of malignant TS-GCT challenging [6].

Bertoni et al. identified common histological features that are found in both primary and secondary malignant TS-GCT, including (1) nodular and infiltrative growth pattern of the synovium and surrounding tissue; (2) large, plump, round, or oval cells with eosinophilic cytoplasm; (3) large nuclei with prominent nucleoli; (4) fewer benign giant cells and xanthomatous cells and inflammatory cells; (5) no maturation between the periphery and the center; and (6) extensive necrosis [6].

Because of the histological variability, the origin of the tumor was unclear. Recently, Al-Ibraheem et al. did extensive immunohotological and cytogenetic analysis of the largest case series of 10 patients and found markers specific for synoviocytes and did not find any markers for macrophages. They concluded that malignant cells are derived from the clusterin-positive large mononuclear cells which represent synoviocytes [7].

We reviewed clinical and radiological features a total 57 cases of malignant tenosynovial giant cell tumor, summarized in Table 1. We found almost equal gender distribution with a slight female predominance (55%). It was found to affect older adults with a median age of 51 years. On rare occasions, it was found in young adults, and only one investigator reported a case in a 12-year-old child with a primary thigh malignant TS-GCT [6]. They were mostly found in the extra-articular soft tissue and with an average size of 9.8 cm found. They tend to present in the lower limb (63%), favoring the soft tissue around the knee, followed by the upper limbs, then axial skeleton. It is notable that both primary and secondary subtypes occurred equally. Secondary malignant recurrence was variable; it occurred either as early as few months or many years following a disease-free period. Kalil and Unni reported secondary malignant transformation 64 years after the initial diagnosis of an ankle TSGCT [8].

Our case showed atypical clinical presentation including younger age of presentation, smaller size, and unusual location. Interestingly, it was found in a subcutaneous location of the midthigh far away from the joint and away from any synovial structure. Such location is strange since it is believed to be synoviocytic in origin [7]. One author reported similar unusual subcutaneous location of malignant TS-GCT one in the gluteal region and another in the upper thigh [24]. Another showed similar subcutaneus location of benign TS-GCT [20]. Our case also showed a considerable smaller tumor size. This feature was also shared with other two reported cases of subcutaneous malignant TS-GCT. This might be due to their superficial location which led to earlier discovery.

MRI features of malignant TS-GCT varied. No specific radiological characteristics were found to differentiate it from other soft tissue sarcoma. Most authors reported malignant TS-GCT as ill-defined heterogenous lobulated lesion. Some found MRI features of classical TS-GCT including presence of dark nodules in both T1 and T2 indicating hemosiderin deposits, multiple lobulation, and periarticular position. Presence of such features may serve as a clue. Areas of necrosis and cystic changes have been also described. Some reported involvement of the underlying bone [17]. Surprisingly, our case showed well defined homogenous enhancement lesion with no lobulation or dark nodules. It was found in the subcutaneous tissue separated from the underlying fascia by a clear fat plane. Such MRI features are uncommon for this tumor.

Initial biopsy showed classical TS-GCT features that might be due to sampling from the benign component. After resection, further analysis showed features of malignant TS-GCT including nested pseudoalveolar proliferation of histiocytes and synoviocytes showing abundant eosinophilic cytoplasm with some perinuclear hemosiderin. Scattered multinucleated osteoclast-like giant cells are seen. The tumor shows atypical cytological features in the form of atypical mitotic figures. The neoplastic cells are diffusely positive for immunohistochemical stain for clusterin antibody.

Malignant TS-GCT was shown to be overly aggressive with high rate of local recurrence and distal metastases. Nearly half of the cases recurred after surgical resection. Some had multiple recurrence and required multiple surgeries. Metastases was also common, with the lung being the most common site in 45% of the cases followed by the regional lymph nodes in 24% of the cases. It also showed tendency to metastasize to other distant and unusual location including spine, pleura, bladder, ilium, mesentery, and thyroid. The unusual preference of spread to the regional lymph node and distal soft tissue sites is another unique feature of this tumor. Prognosis becomes extremely unfavorable with a high mortality rate of 67% once metastases have been discovered.

Surgery is the mainstay of treatment for malignant tenosynovial giant cell tumor [15]. Most investigators treated this disease aggressively radical resection including surgical amputation. Some added additional adjuvant chemotherapy and or radiation therapy. Hence, the discovery of aberrant CSF1 expression and the expression of other markers such as RANK the use of other systemic treatment including denosumab and tyrosin kinase inhibitors with CSF1R inhibitory activity were considered [4, 7, 25]. Due to the small number of cases and the inconsistency of treatment modalities, the efficacy of adjuvant treatment is hard to assess, but did not seem to change the course of the disease. Unfortunately, most cases showed disease progression regardless of mode of treatment with a high tendency for local recurrence and metastasis [4, 15].

## 4. Conclusion

Malignant TS-GCT are extremely rare and difficult to diagnose. Our case showed atypical clinical and radiological features. She was younger than the average, the tumor size was smaller, and the tumor was present in an unusual location in the subcutaneous tissue away from any synovial tissue. In cases with high level of suspension, it is recommended to proceed with biopsy prior to definitive management. The mainstay of treatment in such disease is local surgical control. Other treatment modalities are still under investigation, but so far, it was not able to change the disease course. Due to the aggressive nature of disease and high risk of recurrence and metastases, close follow up is advised.

## Figures and Tables

**Table 1:** Summary of previous reported cases of malignant TS-GCT.

Study	No.	Sex	Age	Location	Type	Size	Type	Recurrence	Metastases	Follow-up
Castens and Howell [3]	1	F	48	Foot	E	NA	Metachronous (6 y)	Yes	Thigh, groin (10 y)	AWD (9 y)

Schajowicz [9]	1	NA	NA	Knee	NA	NA	Metachronous (NA)	No	Lung (2 y)	DOD (2 y)

Ushijima et al. [10]	1	M	59	Knee	I	12	Metachronous (10 y)	Yes	Thigh, LN, back, ribs (2 y)	AWD (6 y)

Nielsen and Kiaer [11]	1	M	67	Knee	I	12	De novo	Yes	Pelvic, sacrum, spine, lung, pleura, thyroid (4 m)	DOD (8 m)

Abdul-Karim et al. [2]	1	F	43	Knee	E	17	Metachronous (11 y)	No	No	NED (5 y)

Shinjo et al. [12]	1	F	72	Hip	I	12	Metachronous (10 m)	Yes	Bladder, pelvic, lung, ilium, mesentery (10 m)	DOD (15 m)

Bertoni et al. [6]	8	M	59	Knee	E	20	De novo	NA	NA	NED (13 y)
		M	57	Foot	E	6	De novo	Yes	Groin (11 m)	DOD (21 m)
		F	37	Knee	NA	NA	Metachronous (10 m)	Yes	No	NED (NA)
		F	74	Knee	NA	NA	De novo	Yes	LN (NA)	DOD (41 m)
		F	71	TMJ	NA	NA	Metachronous (NA)	Yes	Lung (NA)	DOD (NA)
		M	12	Thigh	E	NA	De novo	No	Spine, lung (5 y)	DOD (5 y)
		F	79	Ankle	E	NA	Metachronous (NA)	No	No	DOD (NA)
		F	38	Ankle	I	NA	De novo	No	No	NED (3 y)

Kalil and Unni [8]	1	F	85	Ankle	I	NA	Metachronous (58 y)	Yes	Lung, LN (NA)	DOD (1 y)

Layfield et al. [13]	2	F	65	Knee	I	15	Metachronous (40 y)	Yes	Thigh (NA)	AWD (6 y)
		M	24	Hip	I	15	Metachronous (2 y)	NA	NA	NA

Somerhause and Dal Cin [1]	6	M	42	Hand	E	4.5	De novo	NA	No	NA
		F	21	Thigh	E	3	De novo	Yes	No	NED (13 m)
		F	50	Paravertebral	E	13	De novo	Yes	Lung (21 m)	DOD (35 m)
		F	58	Elbow	E	3	De novo	Yes	NA	NA
		F	33	Sacrococcygeal	E	2.5	De novo	NA	NA	NED (6 m)
		F	26	Knee	E	13	De novo	Nil	Nil	NA

Wu et al. [14]	1	M	27	Forearm	E	1.5	Metachronous (2 m)	No	No	NED (2 y)

Bhadra et al. [15]	3	M	65	Knee	E	4	Metachronous (3 m)	Yes	Pelvis, lung (2 y)	DOD (34 m)
		F	72	Knee	I	NA	Metachronous (8 m)	No	No	NED (6 m)
		F	53	Finger	E	NA	De novo	Yes	No	NED (8 m)

Oda et al. [16]	1	F	53	Sacrum	E	5	Metachronous (10 m)	Yes	LN (7 m)	AWD (NA)
Li et al. [17]	7	M	45	Ankle	I	8	Metachronous (7 m)	No	Spine (9 m)	DOD (NA)
		F	78	Knee	E	8	De novo	Yes	No	NED (NA)
		F	39	Forearm	E	12	Metachronous (31 y)	No	LN, lung (11 m)	AWD (NA)
		M	52	Knee	E	10	De novo	No	No	NED (NA)
		M	68	Elbow	E	6	De novo	No	No	AWD (NA)
		F	67	Leg	E	17	De novo	No	No	NED (NA)
		F	46	Thigh	E	5	De novo	No	LN (9 m)	NED (NA)

Yoon et al. [18]	1	M	29	TMJ	E	7	Metachronous (6 y)	No	Lung (30 m)	AWD (30 m)

Imakiire et al. [19]	1	F	56	Knee	I	NA	Metachronous (11 y)	Yes	Vertebra, pelvis (20 m)	AWD (21 m)

Kondo et al. [20]	1	F	58	Buttock	E	6	De novo	No	Lung (6 m)	AWD (13 m)

Theunissen et al. [21]	1	M	66	Wrist	E	6	Metachronous (2 y)	Yes	No (2 y)	NED (2 y)

Richman et al. [22]	1	F	55	Leg	E	20	Metachronous (7 w)	No	Lung, LN (NA)	DOD (21 m)

Alexiev et al. [23]	1	M	57	Knee	I	4.6	Metachronous (NA)	No	Lung (2 y)	NA

Al-Ibraheemi et al. [7]	10	M	32	Thigh	NA	NA	De novo	No	Lung (NA)	AWD (6 m)
		M	61	Finger	NA	NA	De novo	No	No	NED (15 m)
		M	60	Ankle	NA	NA	Metachronous (NA)	No	Lung, LN (NA)	DOD (6 m)
		F	56	Leg	NA	NA	De novo	No	No	NED (7 m)
		F	72	Thumb	NA	NA	De novo	No	No	NED (5 m)
		M	72	Pelvic	NA	NA	De novo	No	No	DOD (1 m)
		F	26	Toe	NA	NA	De novo	No	No	NED (27 m)
		M	64	Pelvic	NA	NA	Metachronous (NA)	No	Lung (NA)	DOD (66 m)
		M	27	Pelvic	NA	NA	De novo	Yes	No	AWD (48 m)
		M	46	Wrist	NA	NA	De novo	Recent	Recent	Recent

Nakayama et al. [4]	6	F	33	Knee	I	NA	Metachronous (21 y)	Yes	LN, lung (8 m)	DOD (6 y)
		M	53	Thigh	E	NA	De novo	Yes	LN, lung (1 y)	DOD (20 m)
		F	55	Knee	E	NA	Metachronous (3 m)	Yes	LN, lung (2 m)	DOD (23 m)
		F	46	Hip	E	10	De novo	Yes	Lung (initial)	DOD (9 m)
		M	44	Thigh	E	NA	Metachronous (3 y)	Yes	Lung (initial)	DOD (17 m)
		M	55	Knee	NA	NA	Metachronous (3 m)	No	LN, lung (1 y)	DOD (2 y)

Salem et al.	1	F	34	Thigh	E	4.4	De novo	No	No	NED (1 y)

M: male; F: female; NA: not available; E: extra-articular; I: intra-articular; LN: lymph nodes; NED: no evidence of disease; AWD: alive with disease; DOD: death of disease.

## Abbreviations

TS-GCT: Tenosynovial gaint cell tumor.
